# Alterations in high‐dimensional T‐cell profile and gene signature of immune aging in HIV‐infected older adults without viremia

**DOI:** 10.1111/acel.13702

**Published:** 2022-08-29

**Authors:** Min Sun Shin, Hong‐Jai Park, Syim Salahuddin, Ruth R. Montgomery, Brinda Emu, Albert C. Shaw, Insoo Kang

**Affiliations:** ^1^ Department of Internal Medicine Yale University School of Medicine New Haven Connecticut USA

**Keywords:** age, gene expression, human, human immunodeficiency virus (HIV), T cells

## Abstract

Alterations in the components of the immune system occur with aging. The introduction of combination antiretroviral therapy (ART) has dramatically improved life expectancy in human immunodeficiency virus (HIV) infected individuals by suppressing viral replication and increasing CD4^+^ T‐cell counts. Immunosenescence‐like changes, including the expansion of memory CD8^+^ T cells with senescent features, are reported in young HIV‐infected individuals who do not have clinically detectable viremia on ART. However, it is less known whether HIV infection affects the immunosenescent status in older HIV‐infected individuals. Here, we addressed this question in older HIV‐infected, HIV‐uninfected, and frail individuals (all groups age ≥65 years) by examining a set of aging‐associated genes in peripheral blood mononuclear cells (PBMCs) as well as by analyzing subsets of CD4^+^ and CD8^+^ T cells in depth using high‐dimensional CyTOF analysis. Older HIV‐infected individuals had increased expression of aging‐associated genes such as *CX3CR1* in PBMCs which are related to IL‐7 receptor low effector memory (IL‐7Rα^low^ EM) CD8^+^ T cells, a cell population known to expand with age. The subsets of IL‐7Rα^low^ EM CD8^+^ T cells expressing senescent, cytotoxic, and inflammatory molecules, including CD57, perforin, and CX3CR1, as well as memory CD4^+^ T cells expressing CD161 and CXCR3, molecules associated with replication‐competent HIV‐1 harboring cells, were increased in older HIV‐infected individuals. Overall, older HIV‐infected individuals without detectable viremia on ART had augmented levels of age‐associated immune alterations in PBMCs, suggesting that HIV infection has a persistent impact on senescence in older HIV‐infected individuals despite the clinically controlled viremia.

## Graphical abstract

High‐dimensional CyTOF analysis reveals that subsets of IL‐7 receptor alpha low effector memory (IL‐7Rα^low^ EM) CD8^+^ T cells highly expressing cytotoxic, senescent, and inflammatory molecules like perforin, CD57, T‐bet and CX3CR1 expand in order HIV‐infected individuals without viremia on anti‐retroviral therapy compared to control groups. Those findings support the persistence of possible immune senescence driving factor(s) in order HIV‐infected individuals despite the control of viremia.

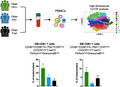

## 1 INTRODUCTION

Aging alters multiple components of the immune system, likely contributing to the increased risk of infections, malignancies, and inflammatory diseases (Lee et al., 2012; Nikolich‐Zugich, 2018). With the marked worldwide expansion of the population over age 60 years, the impact of aging on immune‐related pathologic conditions has become increasingly pronounced. Probably, the two most noticeable age‐associated changes in the immune system are altered proportions of naive and memory T cells, including senescent T cells such as IL‐7 receptor alpha low (IL‐7Rα^low^) effector memory (EM) CD8^+^ T cells, as well as the development of a chronic low‐grade inflammatory status (i.e., inflammaging) with increased circulatory levels of inflammatory molecules like IL‐6 (Lee et al., 2012; Nikolich‐Zugich, 2018; Shaw et al., 2013).

The introduction of combination antiretroviral therapy (ART) has dramatically improved life expectancies in HIV‐infected individuals by suppressing viral replication and improving CD4^+^ T‐cell counts (Wing, 2016). Indeed, over half of the people in the United States with HIV are aged 50 years or older, and this number is expected to increase to 70% by the year 2030 (Wing, 2016). However, studies reported a 6 years gap in life expectancy between HIV‐infected and HIV‐uninfected individuals even after controlling for other factors including ART, smoking, substance abuse, and hepatitis virus infection (Marcus et al., 2016; Marcus et al., 2020). This gap is likely associated with non‐acquired immune deficiency syndrome (non‐AIDS)‐related co‐morbidities such as cardiovascular disease and malignancy (Wing, 2016). Of note, the expected number of comorbidity‐free years remaining at 21 years of age was shorter by 9.5 years in HIV‐infected individuals on ART compared with HIV‐uninfected individuals, supporting the need to prevent comorbidities among individuals with HIV infection (Marcus et al., 2020). Immunosenescence‐like changes including the expansion of memory CD8^+^ T cells with senescent features and increased levels of circulatory inflammatory molecules like IL‐6 are reported in young HIV‐infected individuals without clinically detectable viremia on ART, supporting accelerated immunosenescence in HIV (Desai & Landay, 2010; Regidor et al., 2011; Warren et al., 2019). Though several studies also looked at T cells in HIV‐infected individuals encompassing older people aged ≥65 years on ART (Farhadian et al., 2018; Hove‐Skovsgaard et al., 2020), detailed phenotypic characterization of immune subsets was lacking. It is thus less known whether, and in what manner, HIV infection affects immunosenescent status in older HIV‐infected individuals compared with older HIV‐uninfected individuals.

Here, we investigated the effect of HIV infection on immune aging by analyzing a set of aging‐associated genes in peripheral blood mononuclear cells (PBMCs) as well as by performing high‐dimensional T‐cell analysis in older healthy living HIV‐infected individuals (age ≥65 years) without viremia on ART and control groups, including older HIV‐uninfected healthy living and frail individuals (age ≥65 years). The results of our study show that older HIV‐infected individuals without detectable viremia on ART have augmented levels of age‐associated immune alterations in PBMCs, including aging‐associated gene expression and T‐cell subsets with senescent features, suggesting the persistence of possible senescence driving factors in older HIV‐infected individuals despite viremia control.

## 2 RESULTS

### 2.1 Older HIV‐infected individuals without viremia on ART have increased expression of genes associated with aging and IL‐7Rα^low^ effector memory CD8 ^+^ T cells in PBMC

**FIGURE 1:**
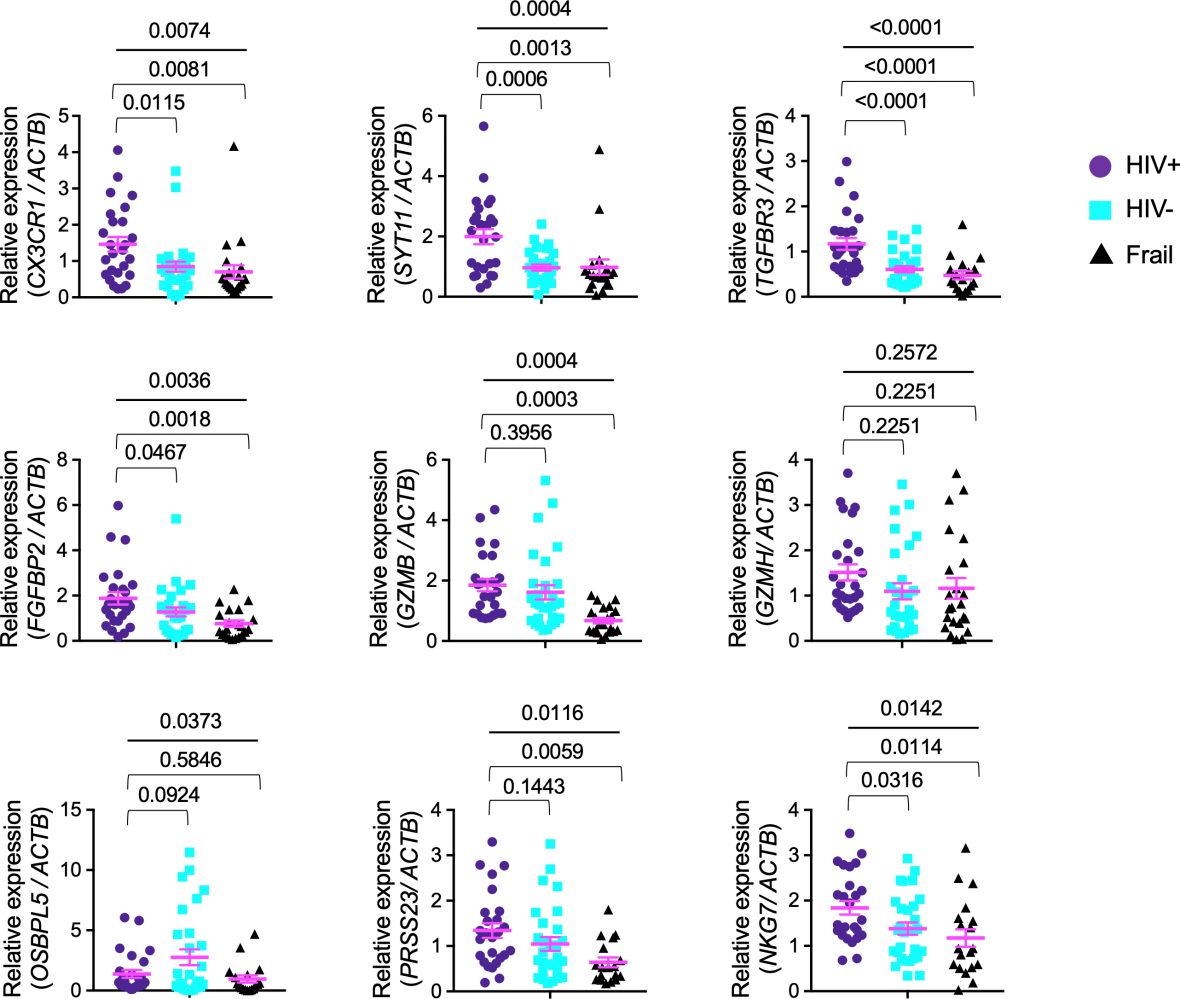
Older HIV‐infected individuals without viremia on antiretroviral therapy (ART) had increased expression of genes associated with aging and IL‐7Rα^low^ effector memory (EM) CD8^+^ T cells in PBMCs. qPCR analysis of indicated genes in PBMCs of older HIV‐infected (HIV+) without viremia on ART, HIV‐uninfected (HIV‐), and frail individuals (all age ≥65 years). Bars indicate the means. *p* values were obtained by one‐way ANOVA with the Holm–Sidak's post hoc test

**FIGURE 2:**
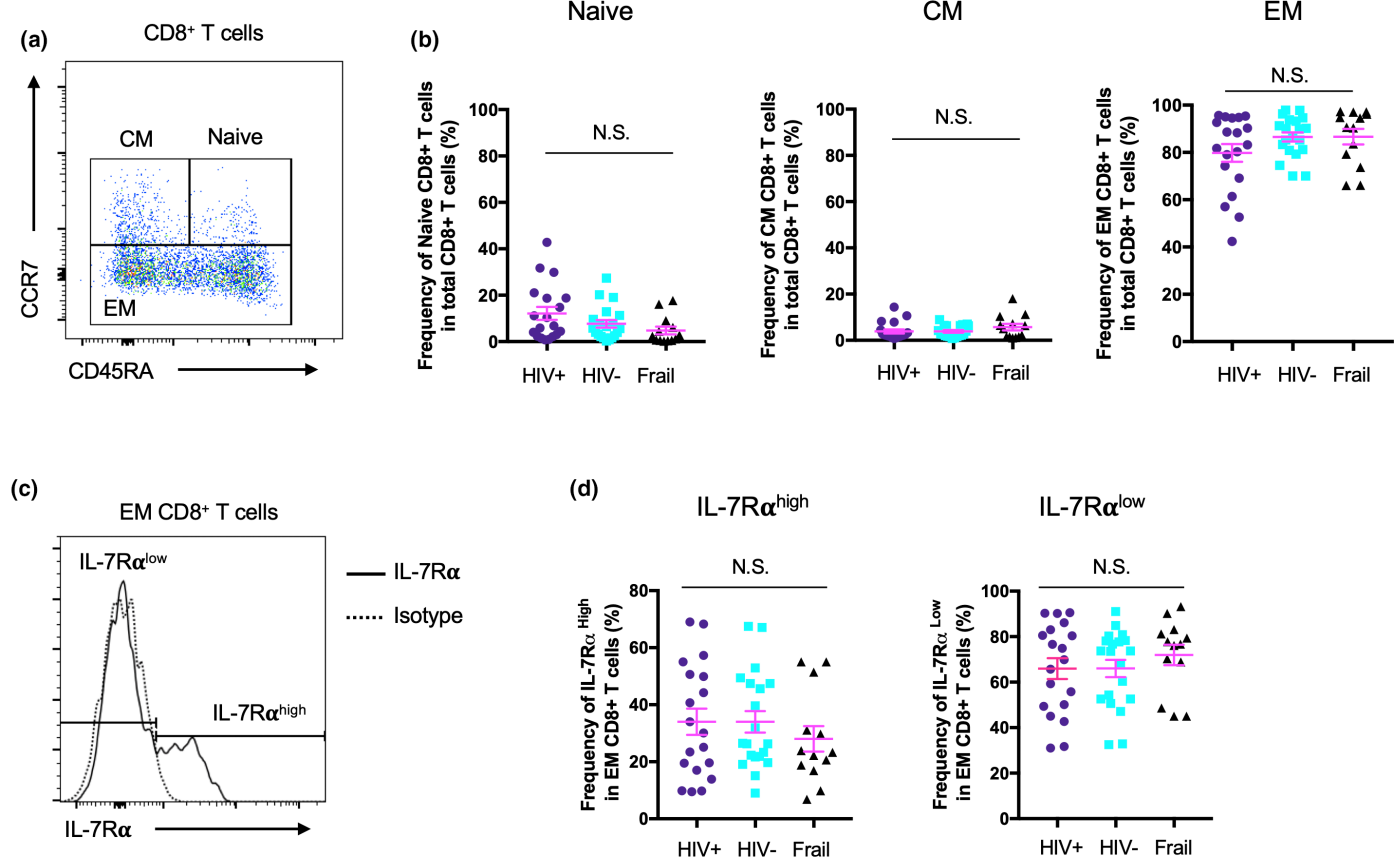
Frequency of IL‐7Rα^low^ effector memory (EM) CD8^+^ T cells was similar in older HIV‐infected without viremia on antiretroviral therapy (ART), HIV‐uninfected, and frail individuals. (a,b) Flow cytometric analysis of naïve (CD45RA^+^ CCR7^+^), central memory (CM, CD45RA^−^ CCR7^+^) and effector memory (EM, CD45RA^+/−^ CCR7^−^) CD8^+^ T cells in PBMCs of older HIV‐infected (HIV+) without viremia on ART, HIV‐uninfected (HIV‐), and frail individuals (age ≥65 years). (a) Representative dot plot and (b) scatter graphs for the frequency of individual cell subsets. (c,d) Flow cytometric analysis of IL‐7Rα^high^ and ^low^ cells in EM CD8^+^ T cells in the same subjects. (c) Representative histogram and (b) scatter graphs for the frequency of IL‐7Rα^high^ and ^low^ cells in EM CD8^+^ T cells. Bars indicate the means. *p* values were obtained by one‐way ANOVA. N.S. indicates non‐significant (*p* values >0.05)

Aging‐associated changes occur in the proportions of naïve and memory T cells including the expansion of senescent IL‐7Rα^low^ EM CD8^+^ T cells in humans (Kim et al., 2006; Lee et al., 2012). A set of 1497 genes associated with chronological age was reported by a meta‐analysis on the global gene expression profile of human peripheral blood from ~15,000 individuals (Peters et al., 2015). We previously found that 231 of these genes, approximately 15% of the 1497 genes, were differentially expressed in IL‐7Rα^low^ EM CD8^+^ T cells (Park et al., 2019). These overlapping genes that were referred to as the aging signature genes of IL‐7Rα^low^ EM CD8^+^ T cells had a strong correlative relationship between log2‐fold changes of IL‐7Rα^low^ versus ^high^ EM CD8^+^ T cells and age‐associated z scores from the meta‐analysis (Park et al., 2019). These findings suggest the relationship of the expansion of IL‐7Rα^low^ EM CD8^+^ T cells with the age‐associated changes in the gene expression profile of peripheral blood cells in non‐HIV‐infected individuals. Of interest, the decreased expression of IL‐7Rα on CD8^+^ T cells was found in HIV‐infected individuals who were not treated with ART, and such a change was partially restored by ART (Colle et al., 2006; MacPherson et al., 2001). We thus explored whether older HIV‐infected individuals without HIV viremia on ART had increased expression of the aging signature genes of IL‐7Rα^low^ EM CD8^+^ T cells, especially nine genes with high z‐scores and fold changes of IL‐7Rα^low^ versus IL‐7Rα^high^ EM CD8^+^ T cells, in peripheral blood cells compared with older HIV‐uninfected and frail individuals. We included older frail individuals, considering whether older HIV‐infected individuals could have immune changes resembling those of older frail individuals who may exhibit substantial loss of physiological reserve. The expression levels of 5 genes, including those encoding the chemokine receptor CX3CR1, the calcium sensing molecule synaptotagmin 11 (SYT11), the TGF‐β receptor 3 (TGFBR3), the cytotoxic cell secreted molecule fibroblast growth factor binding protein 2 (FGFBP2), and the cytotoxic molecule regulator natural killer cell granule protein 7 (NKG7) in PBMCs were higher in older HIV‐infected individuals than in older HIV‐uninfected and frail individuals (Figure 1). Although all the recruited subjects were 65 years or older, we noticed that the mean ages of the 3 groups were dissimilar (mean age ± standard deviation [SD], HIV‐infected 68.2 years ±4.1, HIV‐uninfected 75.7 years ± 7.4, frail 83.9 years ± 11.3, Table 1). The racial proportions of the 3 groups were not similar (Table 1). We thus performed a general linear model analysis to compare the expression levels of the same genes, while controlling for age and race. The increased expression levels of *CX3CR1*, *SYT11,* and *TGFBR3* in HIV‐infected group versus HIV‐uninfected group remained statistically significant (Table S1). The increased expression levels of *FGFBP2* and *NKG7* were also close to the level of statistical significance (*p* = 0.100 and 0.114, respectively, Table 2). In comparison with older HIV‐uninfected individuals, older frail individuals had decreased *GZMB* and *OSBPL5* expression (Table S1). There was no significant relationship of analyzed gene expression levels with years of HIV infection, CD4^+^ T‐cell counts, or history of intravenous (IV) drug use (Table S2, IV drug use data not shown). We next analyzed whether such an increase was secondary to an increased frequency of IL‐7Rα^low^ EM CD8^+^ T cells in older HIV‐infected individuals. The frequency of IL‐7Rα^low^ EM CD8^+^ T cells was similar in older HIV‐infected, HIV‐uninfected, and frail individuals (Figure 2), suggesting that the increased expression of the aging signature genes of IL‐7Rα^low^ EM CD8^+^ T cells in older HIV‐infected individuals is not associated with the proportion of total IL‐7Rα^low^ EM CD8^+^ T cells.

**Table 1:** Characteristics of study subjects

	Age (mean ± SD)	Gender (male: Female)	Race (white: Black: Asian: Others)	CD4^+^ T cell counts (cells/mm^3^) (mean ± SD)	Years of HIV infection (mean ± SD)	Years of anti‐retrovial therapy (mean ± SD)
HIV‐infected individuals^¶^ (*N* = 27^‡^)	68.2 ± 4.1	19:8	5:20:0:2	738.8 ± 248.5	24.1 ± 8.9	20.1 ± 9.3
HIV‐uninfected individuals (*N* = 29^‡^)	75.7 ± 7.4	14:15	23:1:3	NA	NA	NA
HIV‐uninfected frail individuals (*N* = 23^‡^)	83.9 ± 11.3	10:13	23:0:0:0	NA	NA	NA
*p* value	<0.001*	0.241**	<0.001**	NA	NA	NA

^‡^For CyTOF analysis, 12 (HIV+), 15 (HIV‐), and 13 (Frail) subjects from respective groups were studied.

¶8 of 27 subjects had history of intravenous (IV) drug use.

*By one‐way ANOVA; HIV‐infected vs. HIV‐uninfected, *P* = 0.001 by post‐hoc test; HIV‐infected vs. Frail, *P* < 0.001 by post‐hoc test.

**Pearson Chi‐square test

**TABLE 2:** Summary of immune cell alterations in older HIV‐infected individuals

Cell type	*increase in HIV infected vs. uninfected	*decrease in HIV infected vs. uninfected
PBMCs (Figure S2)	Cluster 18: Naïve CD8^+^ T with CD62L^high^ CXCR3^high^ CD27^high^ CD28^high^ CD45RA^high^ CCR7^high^ IL‐7Rα^high^	**Cluster 5: Naïve CD4^+^ T with CD62L^high^ CD27^high^ CD28^high^ CD45RA^high^ CCR7^high^ IL‐7Rα^high^
CD4 (Figure 4)	Cluster 4: CD45RA^low^CCR7^low^CD4^+^ T cells with CD27^low^ CD28^high^ PD‐1^Intermediate (Int)^ CD25^Int^ Cluster 6: CD45RA^low^CCR7^high^CD62L^high^CD4^+^ T cells with CD27^high^ CD28^high^ PD‐1^high^ CXCR3^high^ CXCR5^Intermediate^	
CD8 (Figure 4)	Cluster 12: CD8^+^ T cells with T‐bet^high^ CD56^high^ CD57^high^ perforin^high^ granzyme B^high^ CX3CR1^high^ CD38^low^ IL7Rα^low^ CD27^low^ CD28^low^	
Memory CD4 (Figure 5)	Cluster 5: CD27^low^ CD28^high^ Cluster 10: CD161^high^ PD‐1^high^ CXCR3^high^ CD27^high^ CD28^high^	Cluster 2: CD38^high^ Ki‐67^high^ CD27^high^ CD28^high^ CXCR3^low^
Effector Memory CD8 (Figure 6)	Cluster 3: CD38^high^ CD56^low^ IL‐7Rα^low^ T‐bet^high^ perforin^high^ granzyme B^high^ Cluster 10: CD38^low^ CD56^high^ IL‐7Rα^low^ T‐bet^high^ perforin^high^ granzyme B^high^	

*p values ≤0.05 for all comparisons except **cluster 5 in PBMCs (*p* = 0.0607).

### 2.2 The expression levels of molecules related to T‐cell differentiation and exhaustion by CD4 ^+^ and CD8 ^+^ T cells alter in older HIV‐infected individuals without viremia on ART

**FIGURE 3:**
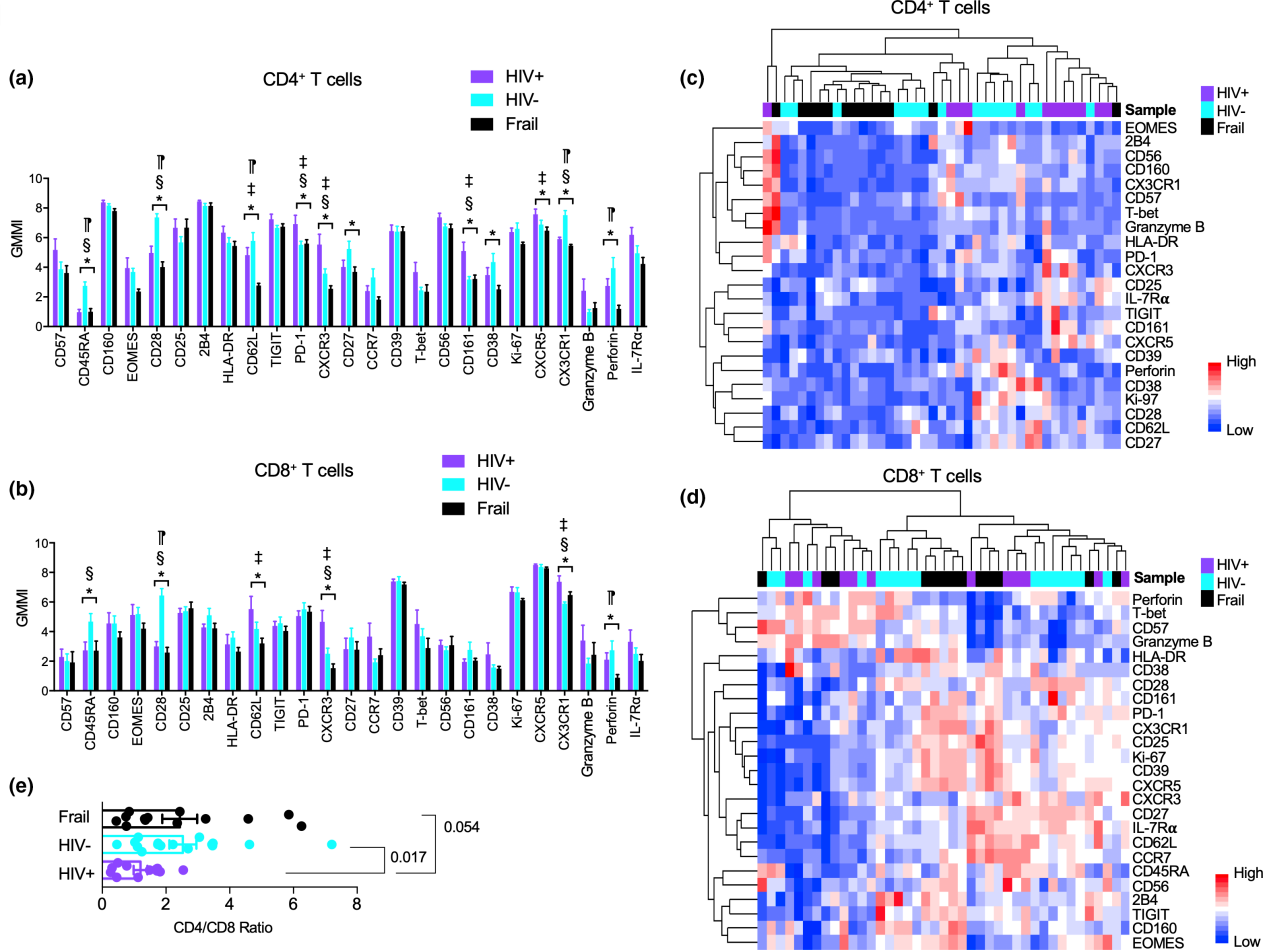
Expression levels of molecules related to T‐cell differentiation and exhaustion by CD4^+^ and CD8^+^ T cells altered in older HIV‐infected individuals without viremia on antiretroviral therapy (ART). (a,b) Expression levels of individual molecules as measured by geometric mean metal intensity (GMMI) in CD4^+^ T and CD8^+^ T cells gated from the acquired CyTOF data of older HIV‐infected (HIV+) (*n* = 12) without viremia on ART, HIV‐uninfected (HIV‐, *n* = 15), and frail (*n* = 13) individuals shown in Figure 3. Bars and error bars indicate the means ± SEM, respectively. *p* values were obtained by one‐way ANOVA with the Holm–Sidak's post hoc test. *p < .05 by one‐way ANOVA. ^§^ *p* < 0.05 HIV+ vs. HIV‐, ^‡^ *p* < 0.05 HIV+ vs. frail, ^⁋^ *p* < 0.05 HIV‐ vs. frail by post hoc analysis. (c,d) Heatmaps showing the results of an unbiased hierarchical clustering analysis of molecules and studied subjects based on the z‐scores of the GMMI of individual molecules

We investigated whether increased expression of the aging signature genes of IL‐7Rα^low^ EM CD8^+^ T cells in older HIV‐infected individuals without viremia on ART could be related to an alteration in the proportions of distinct subsets of IL‐7Rα^low^ EM CD8^+^ T cells since no expansion of total IL‐7Rα^low^ EM CD8^+^ T cells was observed in these individuals. We measured a set of molecules including those for defining immune cell subsets and related to T‐cell activation, differentiation, senescence, effector, and chemotactic function (Table S4) in a total of 40 subjects including older HIV‐infected, HIV‐uninfected, and frail individuals using high dimensional mass cytometry or CyTOF. The mean ages of older HIV‐infected and HIV‐uninfected individuals were similar (mean ± SD, HIV+ 70.0 years ±5.5, HIV‐ 73.8 years ±7.0, *p* = 0.12) although the mean age of older frail individuals was greater than that of older HIV‐infected individuals (83.4 years ±12.4, *p* = 0.002). In analyzing global immune cell populations based on 31 molecules, the proportions of some cell subsets, including T and dendritic cells (DCs), were found to be altered in the studied groups (Figure S2a–c and Table S3). We further explored the possible changes in T‐cell subsets, with an additional analysis of the T‐cell‐associated transcription factors T‐bet and eomesodermin to other molecules. We found altered expression levels of multiple molecules by CD4^+^ and CD8^+^ T cells as determined by geometric mean metal intensities (GMMIs). Consistent with our prior study using flow cytometry as a modality and an independent patient cohort (Farhadian et al., 2018), the GMMIs of CD45RA and CD28, which are known to decrease with T‐cell differentiation, were lower in CD4^+^ and CD8^+^ T cells of older HIV‐infected individuals than in the same cells of older HIV‐uninfected individuals (Figure 3a,b). The GMMIs of these molecules were not different between older HIV‐infected and frail individuals. These findings suggest that the decreased expression of CD45RA and CD28 in older HIV‐infected and frail individuals is likely related to chronic conditions including HIV infection. However, CD4^+^ T cells of older HIV‐infected individuals had increased expression of the T‐cell inhibitory molecule PD‐1 as well as the NK cell associated molecule CD161 (killer cell lectin‐like receptor subfamily B, member 1) compared with both older HIV‐uninfected and frail individuals (Figure 3a) while the expression levels of these molecules by CD8^+^ T cells were not different in the studied groups (Figure 3b). In contrast, the expression levels of CX3CR1 by CD8^+^ T cells were increased in older HIV‐infected individuals compared with older HIV‐uninfected and frail individuals (Figure 3b). Of note, CX3CR1 is highly expressed by cytotoxic and inflammatory IL‐7Rα^low^ EM CD8^+^ T cells which expand with age (Kim et al., 2006; Shin et al., 2015). We tested whether the studied subjects could be clustered based on the expression levels (i.e., GMMI) of individual molecules using an unbiased hierarchical approach. Older HIV‐infected individuals were largely clustered together based on the expression of these molecules by CD4^+^ T cells but not CD8^+^ T cells (Figure 3c,d). The ratio of CD4^+^ to CD8^+^ T cells was lower in older HIV‐infected individuals than in older HIV‐uninfected individuals (Figure 3e, mean ± SD, HIV+ 1.20 ± 0.74 vs. HIV‐ 2.53 ± 1.70, *p* = 0.017), which is in line with the results of previous studies showing low CD4 to CD8 T‐cell ratio in HIV‐infected individuals on ART (Group, 2016; Serrano‐Villar et al., 2014). Our findings indicate that the effect of HIV on the expression of immune molecules by T cells in older individuals is specific for types of cells and molecules although most affected molecules are known to be associated with T‐cell activation, exhaustion, and senescence.

### 2.3 High‐dimensional clustering analysis reveals subsets of CD4 ^+^ and CD8 ^+^ T cells that alter in older HIV‐infected individuals without viremia on ART

**FIGURE 4:**
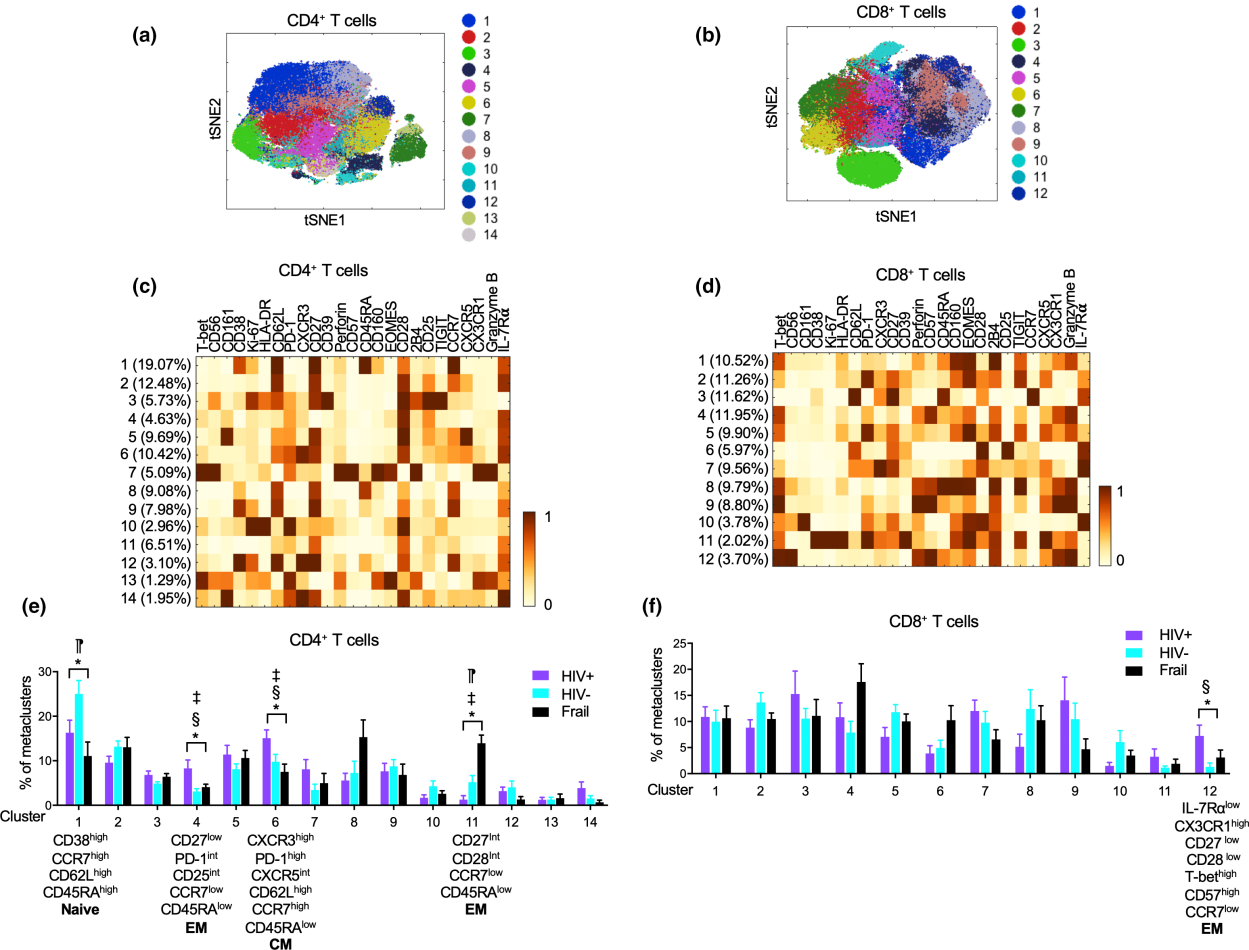
High‐dimensional clustering analysis revealed subsets of CD4^+^ and CD8^+^ T cells that altered in older HIV‐infected individuals without viremia on antiretroviral therapy (ART). *T*‐SNE, PhenoGraph clustering and metaclustering (k parameter set at 15) analyses were performed on pre‐gated CD4^+^ and CD8^+^ T cells of older HIV‐infected (HIV+, *n* = 12) without viremia on ART, HIV‐uninfected (HIV‐, *n* = 15), and frail (*n* = 13) individuals from the acquired CyTOF data shown in Figure 4 (a,b). *T*‐SNE plots showing landscapes of 14 and 12 clusters of CD4^+^ and CD8^+^ T cells, respectively. Numbers and matched color dots indicate individual cell subsets. (c,d) Heatmaps showing the frequency of clusters (Y‐axis) and mean expression levels of indicated molecules (X‐axis) by individual clusters of CD4^+^ (c) and CD8^+^ T cells (d) shown in a and b, respectively. (e,f) Graphs showing the frequency of individual clusters of CD4^+^ (E) and CD8^+^ T cells (f) in older HIV‐infected, HIV‐uninfected, and frail individuals. Bars and error bars indicate the means ± SEM, respectively. *p* values were obtained by one‐way ANOVA with the Holm–Sidak's post hoc test. *p < 0.05 by one‐way ANOVA. ^§^ *p* < 0.05 HIV+ vs. HIV‐, ^‡^ *p* < 0.05 HIV+ vs. frail, ^⁋^ *p* < 0.05 HIV‐ vs. frail by post hoc analysis

We took a clustering approach to further interrogate CD4^+^ and CD8^+^ T‐cell subsets which altered in older HIV‐infected individuals. The frequency of a subset of CD4^+^ T cells with the characteristics of EM T cells (CD45RA^low^CCR7^low^) was increased in older HIV‐infected individuals compared with older HIV‐uninfected and frail individuals (Figure 4a,c and e cluster 4) (see Table 2 for summary). This expanded EM CD4^+^ T‐cell subset had low expression of CD27 but expressed intermediate levels of the T‐cell activation markers PD‐1 and CD25, raising the possible expansion of activated memory CD4^+^ T cells in older HIV‐infected individuals even in the absence of detectable circulatory virus. In CD4^+^ T cells expressing CD62L and CCR7 but not CD45RA, the markers for CM cells, there was a shift towards CXCR3^high^CXCR5^intermediate^PD‐1^high^ cells in older HIV‐infected individuals compared with older HIV‐uninfected and frail individuals (Figure 4a,c and e, cluster 6) (Table 2). Of interest, recent studies reported that circulatory CXCR3^+^ memory CD4^+^ T cells and lymph node PD‐1^+^CXCR5^+^ T follicular helper (Tfh) cells were those with replication‐competent HIV‐1 even in aviremic individuals on ART (Banga et al., 2016; Banga et al., 2018), suggesting the possible detrimental effect of such cell subset expansion in older HIV‐infected individuals without viremia on ART. When analyzing subsets in total CD8^+^ T cells, the frequency of late differentiated senescent and cytotoxic CD8^+^ T cells with low levels of IL‐7Rα, CD27 and CD28, but high levels of T‐bet, CD45RA, CD56, CD57, perforin, granzyme B, 2B4, and CX3CR1, increased in older HIV‐infected individuals compared with older HIV‐uninfected individuals (Figure 4b,d, and f, cluster 12) (Table 2).

**FIGURE 5:**
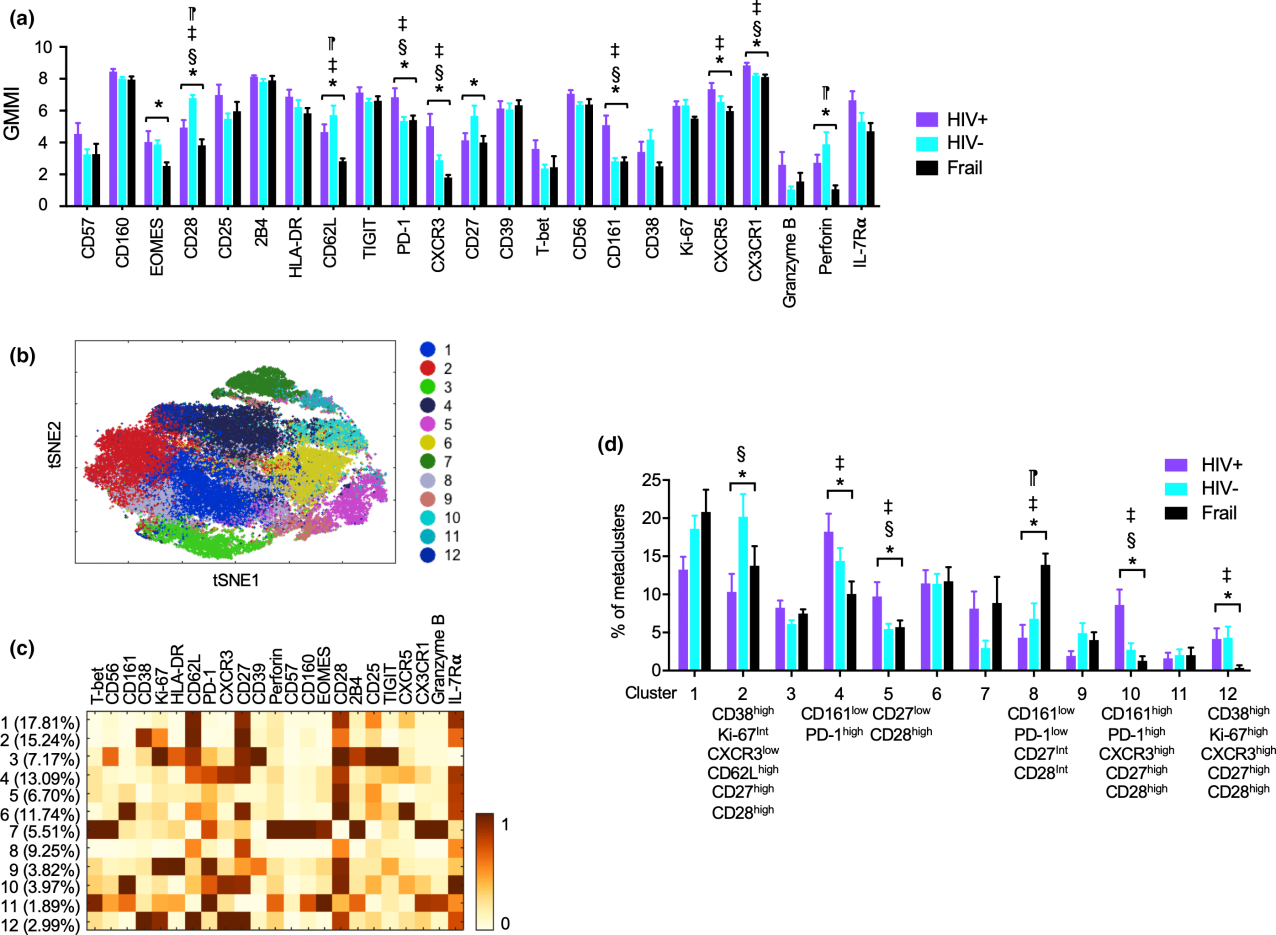
High‐dimensional clustering analysis revealed subsets of memory CD4^+^ T cells that altered in older HIV‐infected individuals without viremia on antiretroviral therapy (ART). (a) Expression levels of individual molecules as measured by geometric mean metal intensity (GMMI) in memory CD4^+^ T cells gated from the acquired CyTOF data of older HIV‐infected (HIV+, *n* = 12) without viremia on ART, HIV‐uninfected (HIV‐, *n* = 15), and frail (*n* = 13) individuals shown in Figure 4. (b‐d) *t*‐SNE, PhenoGraph clustering, and metaclustering (k parameter set at 15) analyses were performed on the memory CD4^+^ T cells based on the expression of molecules indicated by the X‐axis labels in a. (b) *t*‐SNE plot showing landscapes of 12 clusters. Numbers and matched color dots indicate individual cell subsets. (c) Heatmap showing the frequency of clusters (Y‐axis) and mean expression levels of indicated molecules (X‐axis) by individual clusters. (d) Graph showing the frequency of individual clusters in older HIV‐infected, HIV‐uninfected, and frail individuals. Bars and error bars indicate the means ± SEM, respectively. *p* values were obtained by one‐way ANOVA with the Holm–Sidak's post hoc test. *p < 0.05 by one‐way ANOVA. ^§^ *p* < 0.05 HIV+ vs. HIV‐, ^‡^ *p* < 0.05 HIV+ vs. frail, ^⁋^ *p* < 0.05 HIV‐ vs. frail by post hoc analysis

**FIGURE 6:**
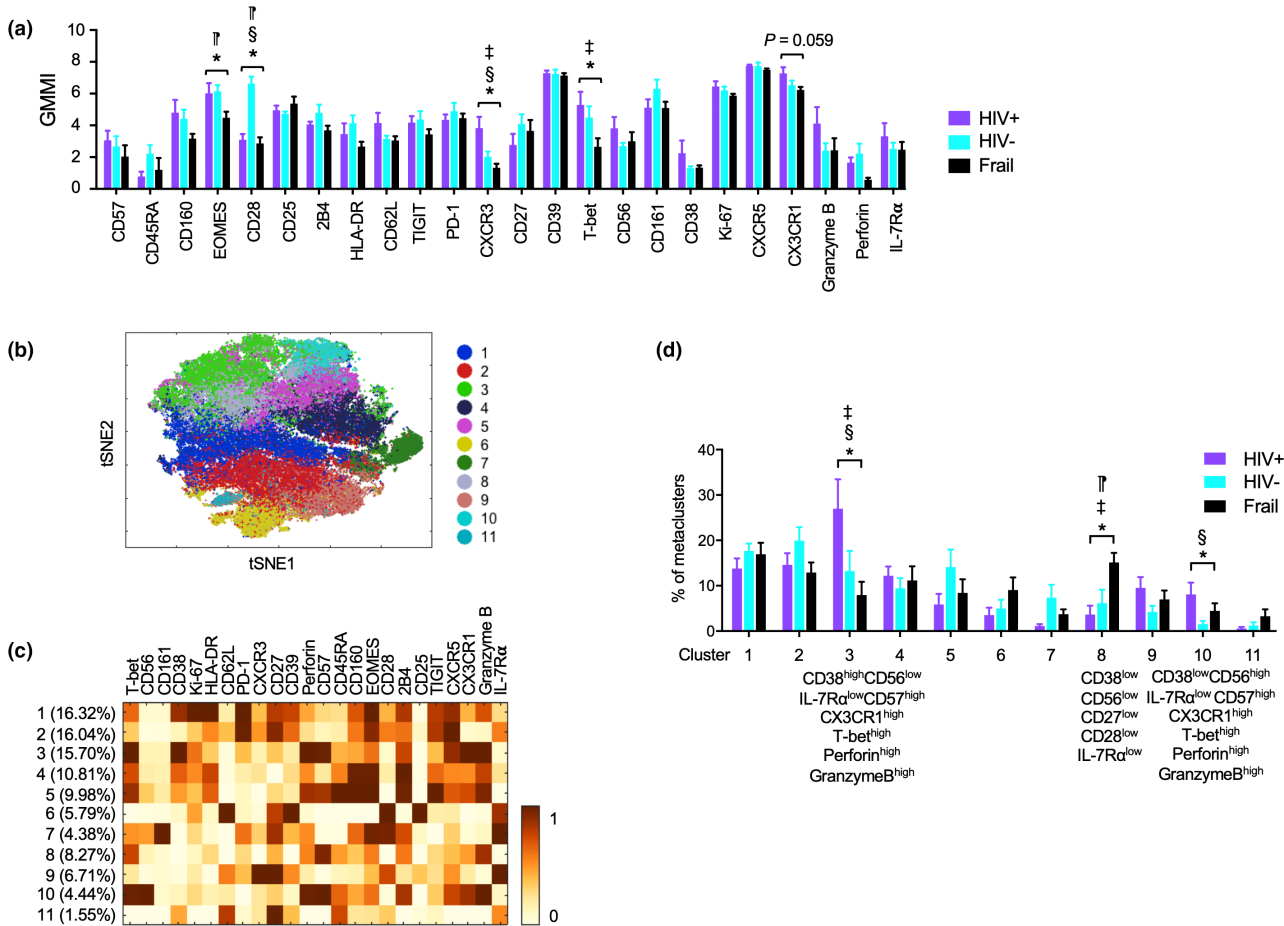
High‐dimensional clustering analysis revealed subsets of effector memory CD8^+^ T cells that altered in older HIV‐infected individuals without viremia on antiretroviral therapy (ART). (a) Expression levels of individual molecules as measured by geometric mean metal intensity (GMMI) in effector memory (EM) CD8^+^ T cells gated from the acquired CyTOF data of older HIV‐infected (HIV+, *n* = 12) without viremia on ART, HIV‐uninfected (HIV‐, *n* = 15), and frail (*n* = 13) individuals shown in Figure 4. (b–d) *t*‐SNE, PhenoGraph clustering, and metaclustering (k parameter set at 15) analyses were performed on the EM CD8^+^ T cells based on the expression of molecules indicated by the X‐axis labels in A. (b) *t*‐SNE plot showing landscapes of 11 clusters. Numbers and matched color dots indicate individual cell subsets. (c) Heatmap showing the frequency of clusters (Y‐axis) and mean expression levels of indicated molecules (X‐axis) by individual clusters. (d) Graph showing the frequency of individual clusters in older HIV‐infected, HIV‐uninfected, and frail individuals. Bars and error bars indicate the means ± SEM, respectively. *p* values were obtained by one‐way ANOVA with the Holm–Sidak's post hoc test. *p < 0.05 by one‐way ANOVA. ^§^ *p* < 0.05 HIV+ vs. HIV‐, ^‡^ *p* < 0.05 HIV+ vs. frail, ^⁋^ *p* < 0.05 HIV‐ vs. frail by post hoc analysis

We further explored the possible effect of HIV on T cells in older individuals by analyzing expression levels of individual molecules by memory CD4^+^ and CD8^+^ T cells as well as by clustering these cells after gating on CD45RA^−^ memory CD4^+^ and CCR7^−^CD45RA^+/−^ EM CD8^+^ T cells based on CCR7 and CD45RA expression. The expression levels of most molecules which changed in total CD4^+^ T and CD8^+^ T cells also altered in such memory cells in older HIV‐infected individuals compared with older HIV‐uninfected and frail groups (Figures 5a and 6a), suggesting the possible changes in the proportions of CD4^+^ and CD8^+^ memory T‐cell subsets in older HIV‐infected individuals. Indeed, older HIV‐infected individuals had a decreased frequency of early differentiated memory CD4^+^ T cells expressing high levels of CD38, CD62L, CD27, and CD28 compared with HIV‐uninfected controls (Figure 5b–d, cluster 2) while the frequency of late differentiated CD27^low^CD28^high^ and CD161^high^PD‐1^high^ memory CD4^+^ T cells increased in older HIV‐infected individuals compared with both control groups (Figure 5b–d, clusters 5 and 10, respectively) (Table 2). Of note, a recent study reported that CD161^+^ CD4^+^ T cells harbored more HIV‐1 DNA and replication‐competent viruses compared with CD161^−^ CD4^+^ T cells (Li et al., 2019). In analyzing EM CD8^+^ T cells, older HIV‐infected individuals had an increased frequency of two subsets of IL‐7Rα^low^ EM CD8^+^ T cells that had similar senescent and cytotoxic phenotypes, including high levels of CD57, perforin, granzyme B, CX3CR1, and T‐bet, except CD38 and CD56 which were highly expressed on respective subsets (Figure 6b–d, clusters 3 and 10, respectively), compared with HIV‐uninfected individuals (Table 2). Older frail individuals had high levels of IL‐7Rα^low^CD27^low^CD28^low^ EM CD8^+^ T cells without the expression of CD38 or CD56 compared with older HIV‐infected individuals (Figures 6b–d, cluster 8). These findings suggest that HIV and frailty have differential effects on CD8^+^ T‐cell senescence. Considering the possible relationship of cytomegalovirus (CMV) infection with immunosenescence, we assessed the frequency of cell clusters in HIV‐infected and HIV‐uninfected individuals who were also CMV infected (CMV+). The frequencies of the CD4^+^ and CD8^+^ T‐cell clusters altered in HIV‐infected individuals were still altered in CMV+ older HIV‐infected individuals compared with CMV+ older HIV‐uninfected individuals, suggesting that HIV‐associated alterations in these T‐cell clusters appears to occur independently of CMV infection (Figure S4). Overall, our high‐dimensional CyTOF analysis indicates that older HIV individuals without viremia on ART have alterations in CD4^+^ and CD8^+^ T‐cell subsets expressing distinct molecules (see Table 2 for summary).

## 3 DISCUSSION

Alterations in the components of the immune system, including cells, organs, and circulating factors, occur with aging (Lee et al., 2012; Nikolich‐Zugich, 2018), likely promoting the risk of infections, malignancy, and inflammatory diseases (Fulop et al., 2011; Goronzy & Weyand, 2005; McElhaney, 2011). Older individuals have altered proportions of naive and memory T cells with the expansion of senescent T cells such as IL‐7Rα^low^ EM CD8^+^ T cells (Kim et al., 2006; Kim, Hwang, & Kang, 2007; Kim, Hwang, Kim, & Kang, 2007; Shin et al., 2015; Shin et al., 2019). Although ART has converted HIV infection from a mortal disease to a chronic controllable condition by dramatically suppressing viral replication (Wing, 2016), immunosenescence‐like changes including the expansion of memory CD8^+^ T cells with senescent features are reported in young HIV‐infected individuals who do not have clinically detectable viremia on ART (Desai & Landay, 2010; Regidor et al., 2011; Warren et al., 2019). Several studies also looked at CD4^+^ and CD8^+^ T‐cell subsets in HIV‐infected individuals encompassing older people (age ≥ 65 years) on ART by analyzing 1–3 molecules related to activation, senescence, and apoptosis (Farhadian et al., 2018; Hove‐Skovsgaard et al., 2020) although detailed phenotypic characterization of immune subsets was lacking. Here, we investigated whether HIV infection affected immunosenescent status in older HIV‐infected individuals as compared with HIV‐uninfected and frail individuals by examining a set of aging‐associated genes in PBMCs as well as by profiling CD4^+^ and CD8^+^ T cells in depth using high‐dimensional CyTOF analysis. The results of our study demonstrated that older HIV‐infected individuals without detectable viremia on ART had augmented levels of age‐associated immune alterations in PBMCs, including aging‐associated gene expression and T‐cell subsets with senescent features, suggesting the persistence of the possible senescence driving factors in older HIV‐infected individuals despite clinically controlled viremia and CD4^+^ T‐cell counts.

In our study, a set of the aging signature genes related to IL‐7Rα^low^ EM CD8^+^ T cells was measured in PBMCs of older HIV‐infected, HIV‐uninfected, and frail individuals. Of the measured genes, *CX3CR1*, *SYT11*, *TGFBR3*, *FGFBP2*, and *NKG7* were increased in the HIV‐infected group compared with the control groups, including older frail individuals, suggesting that the upregulation of these genes is likely related to HIV infection but not to aging alone or general health status. In addition, such a change did not appear to result from the expansion of global IL‐7Rα^low^ EM CD8^+^ T cells since the frequency of global IL‐7Rα^low^ cells in EM CD8^+^ T cells was similar in older HIV‐infected, HIV‐uninfected, and frail individuals as determined by flow cytometry. However, in depth CyTOF analysis identified several subsets of IL‐7Rα^low^ EM CD8^+^ T cells that were altered in HIV‐infected individuals as we had found previously in HIV‐uninfected human subjects using CyTOF and single cell RNA‐seq (Shin et al., 2020). These include IL‐7Rα^low^ EM CD8^+^ T‐cell subsets expressing high levels of the chemokine receptor CX3CR1, the senescent molecule CD57, the cytotoxic molecules perforin and granzyme B, and the transcription factor T‐bet, suggesting a population shift to IL‐7Rα^low^ EM CD8^+^ T cells expressing these molecules in older HIV‐infected individuals. This finding is supported by the results of previous studies showing the expansion of CD57^+^ and CD27^−^CD28^−^ CD8^+^ T cells in HIV‐infected individuals (Simonetta et al., 2014; Tavenier et al., 2015) since IL‐7Rα^low^ EM CD8^+^ T‐cell subsets express low levels of CD27 and CD28. Of note, the expression levels of *CX3CR1* by PBMCs were increased in older HIV‐infected individuals compared with older HIV‐uninfected individuals. In line with our finding, the frequency or percentage of CCR7^−^ (effector memory marker) CD8^+^ T cells expressing CX3CR1 increased in HIV‐infected ART recipients (Mudd et al., 2016). The expansion of highly cytotoxic CX3CR1 expressing IL‐7Rα^low^ EM CD8^+^ T cells may have an implication in HIV‐infected individuals with an increased risk of cardiovascular disease despite ART as these cells can migrate to vascular endothelial and smooth muscle cells through the interaction of CX3CR1 and its ligand, CX3CL1 (fractalkine), which is expressed on activated endothelial cells (Apostolakis & Spandidos, 2013; Damas et al., 2005). In fact, IL‐7Rα^low^ EM CD8^+^ T cells expressing CX3CR1 upregulated CX3CL1 on endothelial cells by producing high levels of IFN‐γ and TNF‐α (Shin et al., 2015), and thrombin induced IFN‐γ release from CX3CR1^high^ EM CD8^+^ T cells (Mudd et al., 2016). Although CM CD8^+^ T cells had relatively low expression levels of CX3CR1, such levels appeared to be higher in older HIV‐infected individuals compared with older HIV‐uninfected and frail individuals (Figure S3). We realize that other immune cells such as NK and CD4^+^ T cells can also express CX3CR1 although we noticed no difference in the frequency of NK cells between older HIV‐infected and HIV‐uninfected individuals.

CD38, also known as cyclic ADP ribose hydrolase, is a transmembrane glycoprotein which is involved in cell adhesion, signal transduction, nicotinamide dinucleotide (NAD+) metabolism, and calcium mobilization (Hogan et al., 2019; Konen et al., 2019). CD38 is considered a T‐cell activation marker and is upregulated on CD8^+^ T cells in untreated HIV‐infected individuals correlating with disease progression (Deeks et al., 2004; Giorgi et al., 2002). However, a decreased frequency of naive T cells expressing CD38 was reported in untreated HIV‐infected individuals compared with ART‐treated patients (Cannizzo et al., 2015). Of interest, we noticed contradictory changes in CD38 expression by memory CD4^+^ and CD8^+^ T cells in older HIV‐infected individuals on ART compared with older HIV‐uninfected individuals. In our study, CD38 expressing EM CD8^+^ T cells could be divided into several subsets, and the expanded CD38 expressing EM CD8^+^ T cell subset in older HIV‐infected individuals was the one with high levels of T‐bet, Ki‐67, perforin, granzyme B, CD57, and CX3CR1 expression but low levels of IL‐7Rα, CD27, and CD28 expression. In memory (CD45RA^−^) CD4^+^ T cells, two CD38^high^ cell subsets that expressed Ki‐67, CD27, CD28, and IL‐7Rα were identified, and the one with low levels of CXCR3 decreased in older HIV‐infected individuals compared with older HIV‐uninfected controls. CD27 and CD28 are down regulated in activated and late differentiated T cells while cytotoxic molecules are upregulated in late differentiated CD8^+^ T cells (Lee et al., 2012). These findings suggest that HIV infection, even in the absence of detectable levels of viremia, can drive the expansion of late differentiated EM CD8^+^ T cells expressing CD38 but reduces early differentiated memory CD4^+^ T cells expressing the same molecule.

We included older frail individuals to assess whether the possible immune changes in older HIV‐infected individuals could also be seen in older frail adults with an age‐associated decline in reserve and function (Xue, 2011). Indeed, CD4^+^ and CD8^+^ T cells of older HIV‐infected and frail individuals had decreased expression of CD45RA and CD28 which are related to T‐cell differentiation and senescence compared with those of older HIV‐uninfected individuals. In contrast, the expression of the checkpoint inhibitory molecule PD‐1 increased on CD4^+^ T cells of older HIV‐infected individuals compared with older uninfected and frail individuals. In addition, 5 and 7 out of the 9 aging associated genes were increased in the peripheral blood of older HIV‐infected individuals as compared with older uninfected and frail individuals, respectively. These findings support the promotion of immunosenescence in older HIV‐infected individuals without viremia on ART who already have a certain degree of immunosenescence secondary to aging. Although the exact mechanism of our findings is yet to be elucidated, these findings could be related to immune stimulation with HIV even in the absence of clinically detectable viremia, presence of co‐infections such as CMV or altered metabolic states, or impact of long‐standing HIV on lymphopoiesis and homeostatic mechanisms. In our study, the immune cell characteristics of older frail individuals had similarities and dissimilarities as compared with older HIV‐infected and uninfected individuals, suggesting the unique feature of immune alterations associated with frailty. Previous studies reported a decreased frequency of peripheral CD28^−^CD8^+^ T cells in older frail individuals compared with older non‐frail individuals (De Fanis et al., 2008; Semba et al., 2005). This finding concurs with the results of our study showing that older frail individuals had decreased CD28 expression by CD4^+^ and CD8^+^ T cells as well as an increased frequency of EM CD8^+^ T cells with low levels of IL‐7Rα, CD27, and CD28 expression as compared with older HIV‐infected and HIV‐uninfected individuals.

In summary, we illustrated the impact of HIV infection on the aging immune system by analyzing a set of aging‐associated genes in PBMCs as well as by performing high‐dimensional T‐cell analysis in older HIV‐infected individuals without viremia on ART, older HIV‐uninfected individuals, and frail individuals. Older HIV‐infected individuals were found to have an expansion of memory CD4^+^ T‐cell subsets including CXCR3^+^CXCR5^+^PD‐1^+^ CM and CD161^+^PD‐1^+^ cells which could contain replication‐competent HIV‐1 even in the setting of aviremia on ART (Banga et al., 2016; Banga et al., 2018; Li et al., 2019). Our results also demonstrated that older HIV‐infected individuals had enhanced expression of a set of genes associated with age and IL‐7Rα^low^ EM CD8^+^ T cells in PBMCs compared with older HIV‐uninfected and frail individuals. In addition, cytotoxic IL‐7Rα^low^ EM CD8^+^ T cells expressing high levels of activation‐, senescence‐, and chemotaxis‐associated molecules CD38, CD56, CD57, and/or CX3CR1 expanded in older HIV‐infected individuals. These findings support that older HIV‐infected individuals without detectable viremia on ART have augmented levels of age‐associated immune alterations in PBMCs, implying the persistence of possible senescence driving factor(s) in older HIV‐infected individuals despite the control of viremia.

## 4 EXPERIMENTAL PROCEDURES

### 4.1 Human subjects

After informed consent, peripheral blood was obtained from HIV‐infected (*n* = 27), HIV‐uninfected (*n* = 29), and frail (*n* = 23) older subjects 65 years of age or older (mean age ± SD, 68.2 years ±4.1, 75.7 years ±7.4, 83.9 years ±11.3, respectively) (clinical characteristics described in Table 1, Tables S5 and S6). The gender distributions were not different among the 3 groups (Table 1). In HIV‐infected subjects, the means of CD4^+^ T‐cell counts, and HIV infection years were 738.8/mm3 and 24.1 years, respectively. All HIV‐infected subjects had CD4^+^ T‐cell counts >200 cells/mm3 and HIV infection years >4 years. The frail status was determined based on the FRAIL scale with 5 components including fatigue, resistance, ambulation, illness, and loss of weight which ranges from 0 to 5 (Abellan van Kan et al., 2008). Frail, pre‐frail, and robust health statuses were defined with scores 3–5, 1–2, and 0, respectively (Morley et al., 2012). In the 23 studied frail subjects, 19 and 4 subjects scored for frail and pre‐frail, respectively. This work was approved by the institutional review committee of Yale University.

### 4.2 Real‐time quantitative reverse‐transcription–polymerase chain reaction (RT‐qPCR)

Peripheral blood mononuclear cells (PBMCs) were purified from blood using FicollPAQUE gradients. Total RNA was isolated from PBMCs using the RNeasy Plus Micro kit (Qiagen, Valencia, CA). Equal amounts of cDNA were synthesized using the Superscript III Reverse Transcriptase Kit (Invitrogen,). qPCR was conducted at 95°C for 5 min, followed by 40 cycles of 95°C for 15 s, 60°C for 30 s, and 72°C for 30 s using the 2x Brilliant SYBR green master mix (Stratagene,) on an Mx3005P QPCR system (Stratagene). Primers used for qPCR (Table S3) were designed using PrimerBank (http://pga.mgh.harvard.edu/primerbank/index.html). The levels of gene expression were normalized to the expression of *ACTINB*. The comparative *C*_T_ method (ΔΔ*C*T) was used for the quantification of gene expression.

### 4.3 Flow cytometry

PBMCs were stained for 30 min at 4°C with antibodies to BUV395–CD3, BV510–CD8α, PE–cyanin 5 (Cy5)–CD45RA, PE–Cy7–CCR7 (all from BD Biosciences), and Pacific Blue–IL–7Rα (Biolegend,). Stained cells were washed with PBS, fixed for 20 min with 2% paraformaldehyde (Biolegend), and acquired (> ~3 × 10^5^ cells) using an LSRII flow cytometer (BD Biosciences). Flow cytometric data were analyzed using FlowJo software (FlowJo, LLC,).

### 4.4 CyTOF analysis

All mass cytometry reagents were purchased from Fluidigm, Inc (South San Francisco,). PBMCs (2 × 10^6^ cells) were stained with a panel of metal‐tagged antibodies (Table S4, including manufacturers) followed by Cisplatin staining. We used the same batches of antibodies for all samples. Cells were first stained with antibodies to surface molecules. For intracellular staining, surface antibody‐stained cells were fixed and permeabilized with Maxpar Fix 1 buffer and Maxpar Perm‐S buffer, respectively, followed by staining with antibodies to intracellular molecules. Stained cells were kept overnight in the MaxPar Fix & Perm Buffer containing intercalator‐Ir. To reduce instrument variation of the Mass Cytometer, stained cells were resuspended in BD FACS lysing solution and stored at −80°C until acquisition on the mass cytometer. Stained cells from all subjects were simultaneously thawed, washed twice with MaxPar Water, resuspended with MaxPar Water containing EQ Four Element Calibration Beads and acquired (>~3 × 10^5^ cells) on the CyTOF2 instrument (Fluidigm). These beads are polystyrene bead standards containing known concentrations of the metal isotopes 140/142Ce, 151/153Eu, 165Ho, and 175/176Lu to normalize mass cytometry data. The data exported as a traditional flow‐cytometry‐file (.FCS) format were normalized using a CYTOF‐preprocessing tool (https://github.com/KrishnaswamyLab/cytof‐preprocessing) and subsequently pre‐gated manually to exclude EQ beads, cell debris, cell doublets, and dead cells using FlowJo software (Figure S1) as previously done (Shin et al., 2019; Shin et al., 2020). The data were transformed using an inverse hyperbolic sine (arcsinh) function with a cofactor of 5. *t*‐SNE, PhenoGraph, and metacluster clustering were done on gated cells (2000 cells) using the CYT, an open‐source analytic tool for CyTOF data (Amir el et al., 2013; Shin et al., 2019; Shin et al., 2020).

### 4.5 Clustering and principal component analyses

Unbiased hierarchical clustering analysis was conducted using the publicly available R‐based Pheatmap (version 1.0.12) packages.

### 4.6 Statistical analysis

The one‐way ANOVA test was used to compare difference in gene expression, geometric mean metal intensity (GMMI), and cell frequency across the different study groups with controlling multiple comparisons as indicated in the figure legends using the Holm–Sidak's post hoc test. Categorical variables were compared using the Pearson Chi‐square test. In addition, the general linear model was used to control for age and race in comparing gene expression in the study groups. Statistical analysis was done using the IBM SPSS 28.0 and GraphPad Prism 8. *p* < 0.05 was considered statistically significant.

## AUTHOR CONTRIBUTIONS

MS and IK designed and performed experiments, analyzed data, and wrote the manuscript. HP and SS performed experiments. BE, ACS, and RRM contributed to experimental design, discussions, and/or provided intellectual input.

## FUNDING INFORMATION

This work was supported in part by grants from the National Institutes of Health (R01 AG055362 to IK and ACS; R01 AG056728 to IK; K24 AG042489 to ACS; and AI089992 to RRM and ACS).

## CONFLICT OF INTEREST

The authors declare no competing financial interests.

## Supporting information


Figure S1‐S4

Table S1‐S6

## Data Availability

The data that support the findings of this study are available from the corresponding author upon reasonable request.
