# Supplementary material for: Alterations in high‐dimensional T‐cell profile and gene signature of immune aging in HIV‐infected older adults without viremia
Source: Aging Cell. 2022 Aug 29;21(10):e13702. doi: 10.1111/acel.13702 (PMC9577958; doi:10.1111/acel.13702)
Supplement: Supplementary file 1 — Figure S1‐S4 [file ACEL-21-e13702-s002.pdf]

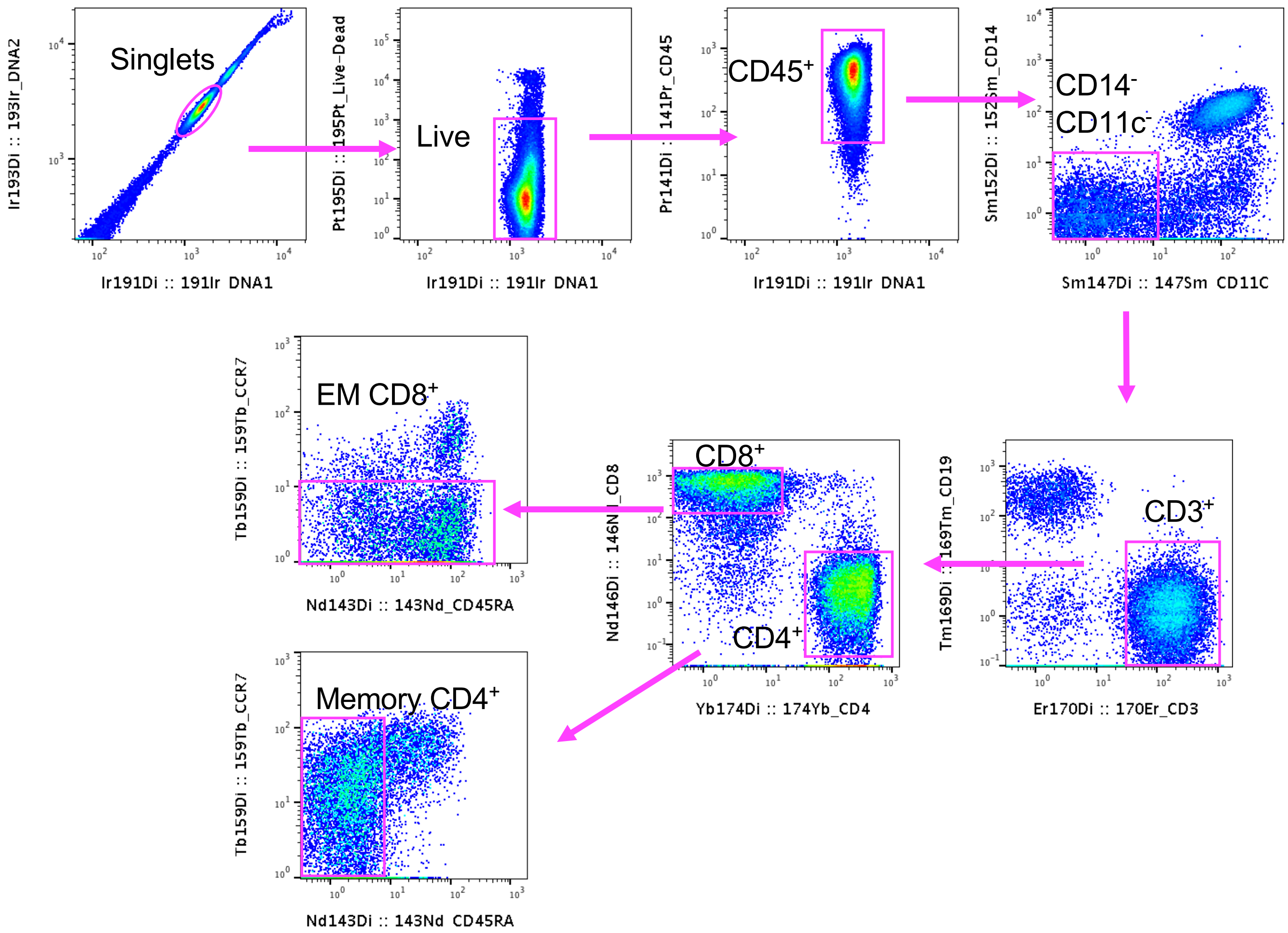

Supplementary Fig. 1 Representative gating strategy for identifying immune cell subsets from mass cytometry datasets. PBMCs isolated freshly from a donor were stained with the indicated antibodies. Frist, cell doublets and dead cells were excluded and CD45^{+} leukocytes were identified. Subsequent gates were designed to isolate CD4^{+} T cells (CD19-CD14-CD11c-CD3+CD4+) and CD8^{+} T cells (CD19-CD14-CD11c-CD3+CD8+). Within these cell subsets, memory CD4^{+} T cells and effector (EM) CD8^{+} T cells were identified based on the expression of CCR7 and CD45RA.

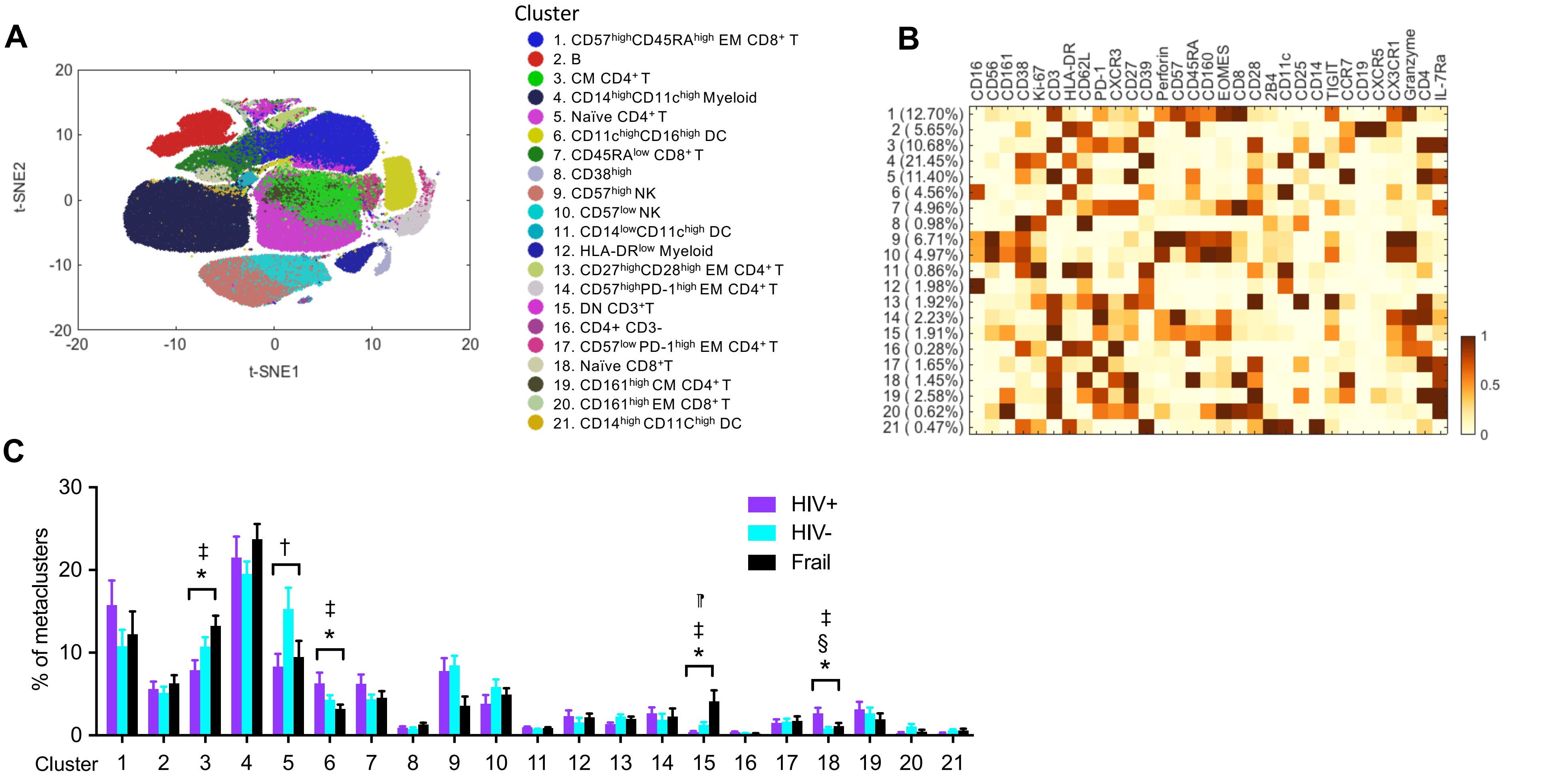

Supplementary Fig. 2 Global immune cell subsets alter in older HIV-infected individuals without viremia on antiretroviral therapy (ART) as determined by high-dimensional CyTOF analysis. PBMCs of older HIV-infected (HIV+, *n* = 12) without viremia on ART, HIV-uninfected (HIV-, *n* = 15), and frail (*n* = 13) individuals (age ≥ 65 years) were stained with antibodies to a set of molecules (Supplementary Table 3) and run on a CyTOF2 instrument. *t*-SNE, PhenoGraph clustering and metaclustering (k parameter set at 15) analyses were performed on PBMCs based on the expression of 31 molecules indicated by the X-axis labels in (B). (A) *t*-SNE plot showing a landscape of 21 clusters. Numbers and matched color dots indicate individual cell subsets. (B) Heatmap showing the frequency of clusters (Y-axis) shown in (A) and mean expression levels of 31 molecules (X-axis) by individual clusters. (C) Graph showing the frequency of individual clusters in older HIV+, HIV- and frail individuals (see cluster legends in (A) for cluster annotations). Bars and error bars indicate the means ± SEM, respectively. *P* values were obtained by one-way ANOVA with post hoc analysis test. *$P < 0.05$ by one-way ANOVA. §$P < 0.05$ HIV+ vs. HIV-, ‡$P < 0.05$ HIV+ vs. Frail ¶$P < 0.05$ HIV- vs. Frail by the Holm-Sidak's post-hoc test. †$P = 0.0585$ by one-way ANOVA. EM, effector memory; CM, central memory; DC, dendritic cells; NK, natural killer cells, DN, double negative.

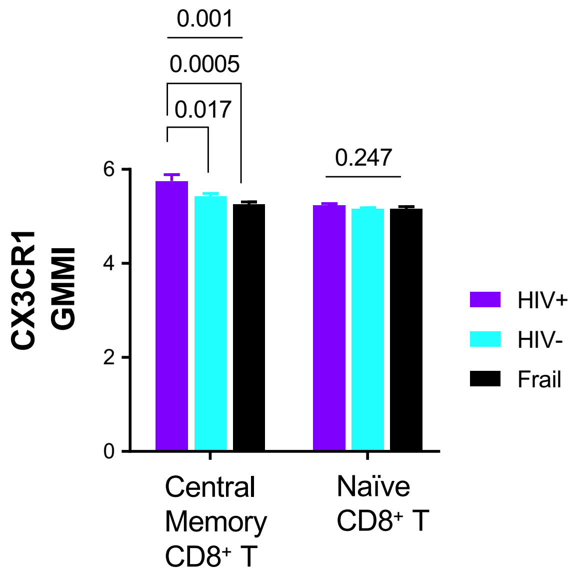

Supplementary Fig. 3 The Expression levels of CX3CR1 as measured by geometric mean metal intensity (GMMI) in central memory and naïve $CD8^+$ T cell subsets gated from the acquired CyTOF data of older HIV-infected (HIV+) (*n* = 12) without viremia on ART, HIV-uninfected (HIV-, *n* = 15), and frail (*n* = 13) individuals shown in Figure 3. Bars and error bars indicate the means ± SEM, respectively. *P* values were obtained by one-way ANOVA with the Holm-Sidak's post-hoc test.

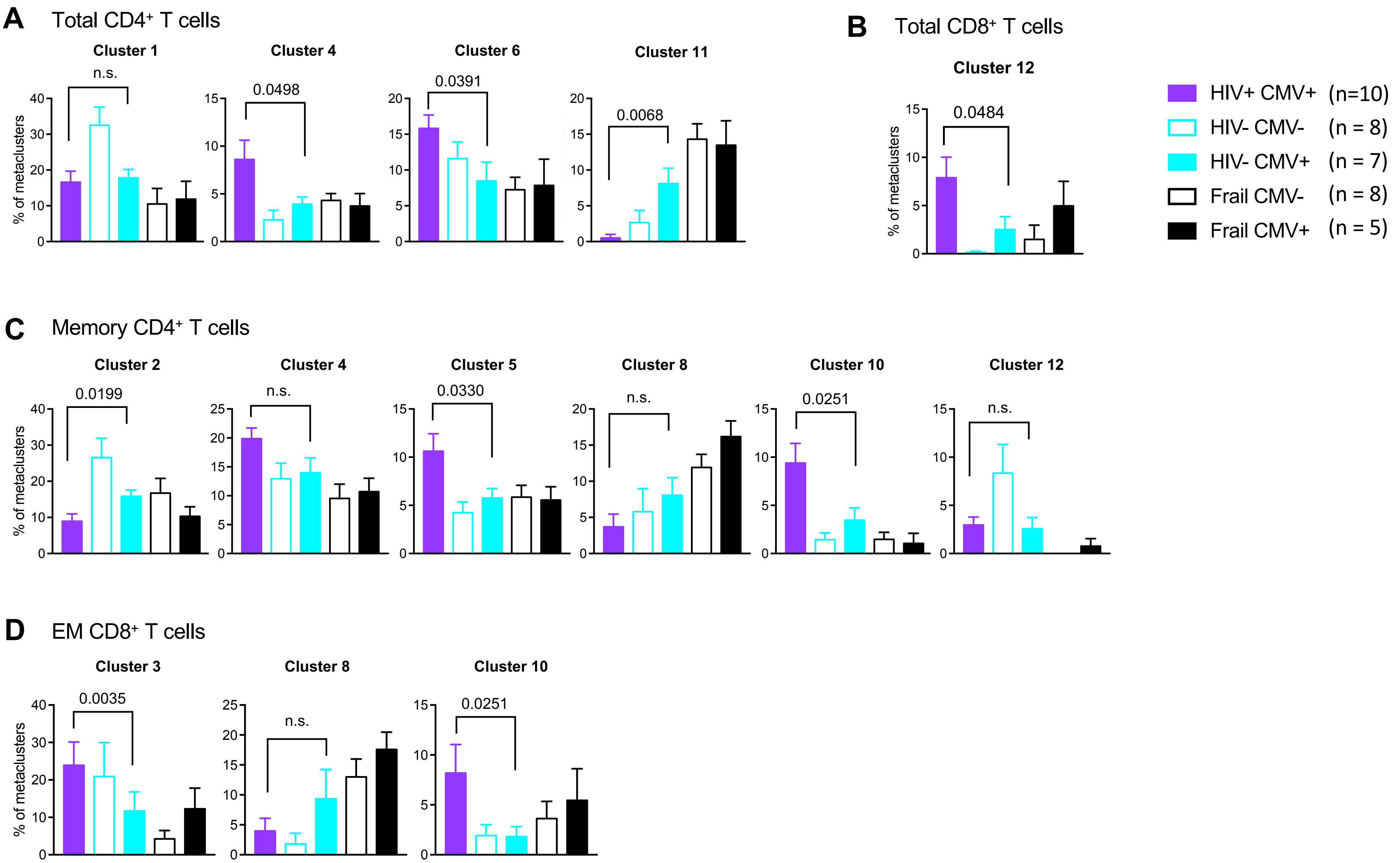

Supplementary Fig. 4 The altered frequencies of T cell subsets in older HIV-infected individuals without viremia on ART occur independently of cytomegalovirus (CMV) infection. (A-B) The frequencies of CD4$^+$ T and CD8$^+$ T cell clusters shown in Figure 4 in older HIV-infected, HIV-uninfected, and frail individuals who were CMV-infected (CMV+) or CMV-uninfected (CMV-). (C-D) The frequencies of memory CD4$^+$ T and EM CD8$^+$ T cell clusters shown in Figure 5 in older HIV-infected, HIV-uninfected, and frail individuals who were CMV+ or CMV-. Bars and error bars indicate the means ± SEM, respectively. *P* values were obtained by unpaired *t*-test with the Welch's correction (HIV+ CMV+ vs. HIV- CMV+). n.s. indicates non-significant (*P* values > 0.05).
