# Supplementary material for: Alterations in high‐dimensional T‐cell profile and gene signature of immune aging in HIV‐infected older adults without viremia
Source: Aging Cell. 2022 Aug 29;21(10):e13702. doi: 10.1111/acel.13702 (PMC9577958; doi:10.1111/acel.13702)
Supplement: Supplementary file 2 — Table S1‐S6 [file ACEL-21-e13702-s001.docx]

**Supplementary Table 1**. **P* values obtained by comparing 9 aging signature genes of IL-7Rα^low^ effector memory CD8^+^ T cells in PBMCs of older HIV-infected, -uninfected and frail individuals using the general linear model controlling for age and race.

| **Gene** | **HIV infected vs. uninfected** | **HIV infected vs. frail** | **HIV uninfected vs. frail** |
| --- | --- | --- | --- |
| *CX3CR1* | 0.035 | 0.014 | 0.920 |
| *SYT11* | 0.005 | 0.014 | 1.000 |
| *TGFBR3* | 0.002 | <0.001 | 0.811 |
| *FGFBP2* | 0.100 | 0.002 | 0.230 |
| *GZMB* | 0.798 | 0.001 | 0.009 |
| *GZMH* | 0.332 | 0.529 | 0.993 |
| *OSBPL5* | 0.051 | 0.875 | 0.017 |
| *PRSS23* | 0.429 | 0.018 | 0.255 |
| *NKG7* | 0.114 | 0.026 | 0.762 |

**P* values by post hoc Sidak (2 sided) analysis

**Supplementary Table 2. Correlation of gene expression levels with CD4^+^ T cell counts and HIV infection years**

|  |  | CD4^+^ T cell count | HIV infected years |
| --- | --- | --- | --- |
| *CX3CR1* | *r* | -0.168 | 0.283 |
|  | *P* | 0.412 | 0.161 |
| *NKG7* | *r* | -0.119 | 0.332 |
|  | *P* | 0.564 | 0.098 |
| *FGFBP2* | *r* | 0.126 | 0.275 |
|  | *P* | 0.538 | 0.174 |
| *GZMB* | *r* | 0.009 | 0.246 |
|  | *P* | 0.964 | 0.227 |
| *GZMH* | *r* | -0.041 | 0.178 |
|  | *P* | 0.841 | 0.383 |
| *OSBPL5* | *r* | -0.079 | 0.112 |
|  | *P* | 0.702 | 0.585 |
| *SYT11* | *r* | 0.013 | 0.078 |
|  | *P* | 0.952 | 0.706 |
| *TGFBR3* | *r* | 0.234 | 0.082 |
|  | *P* | 0.249 | 0.691 |
| *PRSS23* | *r* | 0.117 | 0.2 |
|  | *P* | 0.571 | 0.326 |

*r*: correlation coefficient

*P*: *P* values by Pearson correlation analysis

**Supplementary Table 3.** Primer sequences for qPCR

| **Gene** | **Primer sequence** | **Annealing temp (°C)** |
| --- | --- | --- |
| *CX3CR1* | For: 5'-TTGCCCTCACCAACAGCAAG-3' | 60°C |
|  | Rev: 5'-AAGGCGGTAGTGAATTTGCAC-3' |  |
| *SYT11* | For: 5'-GGGAAGGTGGACGTAGGAAC-3' | 60°C |
|  | Rev: 5'-GGGGTCAGGCTTGTAATAGGG-3' |  |
| *TGFBR3* | For: 5'-TGGGGTCTCCAGACTGTTTTT-3' | 60°C |
|  | Rev: 5'-CTGCTCCATACTCTTTTCGGGG-3' |  |
| *FGFBP2* | For: 5'-TTCCTGCACTATGCGTCCC-3' | 60°C |
|  | Rev: 5'-GGGCTTGATTCCAGTAAGGTTT-3' |  |
| *NKG7* | For: 5'-CATCATATCAGGCTACATCCACG-3' | 60°C |
|  | Rev: 5'-GCTGCGGTGGTTGAGACAA-3' |  |
| *GZMH* | For: 5'-ATGAGCACTTTAGCAACCACAC-3' | 60°C |
|  | Rev: 5'-CGGAGTCCCCCTTGAAACC-3' |  |
| *GZMB* | For: 5'GGGGACCCAGAGATTAAAAA-3' | 60°C |
|  | Rev: 5'-GCTCCAGAGAAGGTGTTTCA-3' |  |
| *OSBPL5* | For: 5'-CTCCGAGTCAGATGGTCGC-3' | 60°C |
|  | Rev: 5'-TGCCCAGTCTCAGTAGGCT-3' |  |
| *PRSS23* | For :5'-GGAAGCGGCAGATTTATGGCT-3' | 60°C |
|  | Rev: 5'-TTCCATCGTGTATGCAGTGGG-3' |  |
| *ACTINB* | For: 5'-CGTGGACATCCGCAAAGAC-3' | 60°C |
|  | Rev: 5'-TGCATCCTGTCGGCAATG-3' |  |

**Supplementary Table 4.** Panels of antibodies, including metal-tagged ones, against cell surface markers and intracellular molecules.

| **Antibody** | **Type** | **Antibody clone** | **Isotope/ Fluorochrome** | **Supplier** |
| --- | --- | --- | --- | --- |
| EOMES | Transcription factor | WD1928 | PE | eBiosciences |
| anti-PE | Secondary antibodies | PE001 | 145Nd | Fluidigm |
| CD45 | Hematopoietic cell marker | HI30 | 141Pr | Fluidigm |
| CD57 | T cell senescent marker | HCD57 | 142Nd | Fluidigm |
| CD45RA | Naïve/Memory T cell marker | HI100 | 143Nd | Fluidigm |
| CD160 | T cell exhaustion | By55 | 144Nd | HMA CyTOF Antibody Resource |
| CD8 | CD8 T cell marker | RPA-T8 | 146Nd | Fluidigm |
| CD11c | Dendritic cell marker | Bu15 | 147Sm | Fluidigm |
| CD28 | Co-stimulatory molecules | CD28.2 | 148Nd | HMA CyTOF Antibody Resource |
| CD25 | IL-2 receptor | 2A3 | 149Sm | Fluidigm |
| CD244 (2B4) | T cell exhaustion | C1.7 | 150Nd | HMA CyTOF Antibody Resource |
| HLA-DR | MHC class II | G46-6 | 151Eu | Fluidigm |
| CD14 | Monocyte marker | M5E2 | 152Sm | HMA CyTOF Antibody Resource |
| CD62L | T cell activation | DREG-56 | 153Eu | Fluidigm |
| TIGIT | T cell exhaustion | MBSA43 | 154Sm | Fluidigm |
| PD-1 | T cell exhaustion | EH12.2H7 | 155Gd | Fluidigm |
| CXCR3 | Chemokine receptor | G025H7 | 156Gd | Fluidigm |
| CD27 | Co-stimulatory molecules | L128 | 158Gd | Fluidigm |
| CCR7 | Naïve/Memory T cell marker | G043H7 | 159Tb | Fluidigm |
| CD39 | T cell exhaustion | A1 | 160Gd | Fluidigm |
| T-bet | Transcription factor | 4B10 | 161Dy | Fluidigm |
| CD56 | Natural killer cell marker | NCAM16.2 | 163Dy | Fluidigm |
| CD161 | Cytotoxic memory cell marker | HP-3G10 | 164Dy | Fluidigm |
| CD38 | Activation marker | HIT2 | 167Er | Fluidigm |
| Ki-67 | Proliferation | Ki67 | 168Er | Fluidigm |
| CD19 | B cell marker | HIB19 | 169Tm | Fluidigm |
| CD3 | T cell maker | UCHT1 | 170Er | Fluidigm |
| CXCR5 | Chemokine receptor | 51505 | 171Yb | Fluidigm |
| CX3CR1 | Chemokine receptor | 2A9-1 | 172Yb | Fluidigm |
| Granzyme B | Cytotoxic molecules | GB11 | 173Yb | Fluidigm |
| CD4 | CD4 T cell maker | SK3 | 174Yb | Fluidigm |
| Perforin | Cytotoxic molecules | B-D48 | 175Lu | Fluidigm |
| IL-7Ra | Cytokine receptor | A019D5 | 176Yb | Fluidigm |
| CD16 | Natural killer cell marker | 3G8 | 209Bi | Fluidigm |

**Supplementary Table 5*.** Summary of common comorbid conditions**

|  | HIV-infected individuals (n = 27) | HIV-uninfected individuals (n = 29) | HIV-uninfected frail individuals (n = 23) |
| --- | --- | --- | --- |
| Hypertension | 15 | 14 | 16 |
| Diabetes mellitus | 8 | 3 | 10 |
| Coronary artery disease | 5 | 2 | 6 |
| Congestive heart failure | 4 | 1 | 8 |
| Stroke/Transient ischemic attack | 3 | 2 | 6 |
| Hemiplegia | 0 | 0 | 4 |
| Chronic pulmonary disease | 2 | 3 | 4 |
| Renal insufficiency | 1 | 0 | 7 |
| Thyroid disease | 0 | 3 | 5 |

*Numbers indicate the numbers of positive cases for individual conditions

**>5% of subjects of any group were affected.

**Supplementary Table 6*.** Antiretroviral medications taken by HIV-infected individuals

| darunavir | ritonavir | abacavir | lamivudine | dolutegravir | efavirenz | emtricitabine | tenofovir | atazanavir | bictegravir | rilpivirine | cobicistat |
| --- | --- | --- | --- | --- | --- | --- | --- | --- | --- | --- | --- |
| 4 | 3 | 3 | 3 | 6 | 2 | 17 | 17 | 1 | 8 | 4 | 2 |

*numbers indicate the numbers of HIV-infected subjects (n=27) on individual medications.
